# Non-invasive imaging in acute and chronic pulmonary embolism

**DOI:** 10.1093/bjro/tzaf005

**Published:** 2025-04-10

**Authors:** Sze Mun Mak, Bhavin Rawal, Giulia Benedetti, Amy Eccles, Laura Price, Karen Breen, Simon P G Padley, Narayan Karunanithy

**Affiliations:** Department of Radiology, Guy’s and St Thomas’ NHS Foundation Trust, London, SE1 7EH, United Kingdom; Cardiovascular Imaging Department, King’s College London, St Thomas Campus, London, SE1 7AR, United Kingdom; Department of Radiology, Royal Brompton & Harefield NHS Foundation Trust, London, SW3 6NP, United Kingdom; Department of Radiology, Guy’s and St Thomas’ NHS Foundation Trust, London, SE1 7EH, United Kingdom; Department of Radiology, Guy’s and St Thomas’ NHS Foundation Trust, London, SE1 7EH, United Kingdom; National Pulmonary Hypertension Service, Royal Brompton & Harefield NHS Foundation Trust, London, Uxbridge, UB9 6JH, United Kingdom; Thrombosis & Haemophilia Centre, Guy’s and St Thomas’ NHS Foundation Trust, London, SE1 7EH, United Kingdom; Department of Radiology, Royal Brompton & Harefield NHS Foundation Trust, London, SW3 6NP, United Kingdom; Department of Radiology, Guy’s and St Thomas’ NHS Foundation Trust, London, SE1 7EH, United Kingdom

**Keywords:** pulmonary emboli, risk stratification, CT pulmonary angiogram, chronic thromboembolic pulmonary hypertension

## Abstract

Acute pulmonary emboli can manifest as a spectrum of physiological status ranging from an incidental finding to life threatening right heart failure. We discuss the crucial role imaging plays in the accurate and rapid diagnosis. In addition, imaging features are central in assessing the severity of the presentation allowing for appropriate risk stratification and escalation of care. The relative strengths of the various imaging modalities used in the management of chronic thromboembolic pulmonary hypertension are also discussed.

## Introduction 

### Background

Pulmonary embolism (PE) is defined as a complete or partial obstruction of a pulmonary artery usually caused by thrombus. Occasionally emboli are composed of tumour fragments, gas locules or globules of fat. PE is the commonest presentation of venous thromboembolism (VTE) together with deep vein thrombosis (DVT). PE can be lethal in the acute phase if the burden of thrombus is sufficient. Incomplete resolution of thrombus can result in the development of chronic thromboembolic disease (CTED), where pulmonary artery pressures are normal at rest, or chronic thromboembolic pulmonary hypertension (CTEPH) where pulmonary artery pressures are elevated at rest. Both are associated with high morbidity and mortality.^1^

The reported incidence of VTE is 100–200 cases per 100 000 of the population making it the third most common cardiovascular disease after acute myocardial infarction and stroke.^2^ The WHO has estimated that annually there are 10 million cases of VTE associated with hospital admission.^2^ The incidence of PE as a result of VTE is estimated to be approximately 60–70 cases per 100 000.^3^ In the UK, 47 594 cases of PE were reported in the 1-year period between 2013 and 2014 and accounted for nearly 28 000 hospital admissions and 250 000 bed days in 2011.^3^ However, these figures do not include PE occurring in patients already in hospital.

### Aetiology

VTE is associated with both hereditary and acquired risk factors. Hereditary risk factors for VTE include factor V Leiden mutation, mutations of prothrombin G20210A, deficiency of protein S and C.^4^ Acquired risk factors can be sub-classified as provoking or non-provoking. Provoking risk factors include recent surgery, trauma, immobilization, pregnancy, hospital admission, infection, and active cancer whereas non-provoking risk factors include obesity and cigarette smoking.^5^ It is theorized that some pulmonary artery thrombus develops *in situ* rather than being embolized from elsewhere, a phenomenon particularly associated with the COVID-19 pandemic. This is thought to be due to intense inflammation and endothelial dysfunction from pneumonitis.^6^

The most common sources of PE (up to 85% of cases) are DVT of the lower limbs or pelvis followed by thrombosis of renal veins and the inferior vena cava. Most DVTs arise from femoral and popliteal veins and more than 50% of patients with DVT in these segments will have concurrent PE at presentation.^7^ Calf vein DVT rarely embolize to the lungs and majority of calf vein thrombi resolve spontaneously. The upper limbs are an unusual source of major PE but are associated with indwelling long-term venous catheters and pacemakers.

### Physiological consequences of acute PE

In the presence of PE, the affected lung tissue is still ventilated. However reduced or absent perfusion results in impaired gas exchange. Pulmonary infarction may occur if there is no collateral perfusion.^1^ It is not usually until PE occludes 30–50% of the total cross section of the pulmonary arterial bed that pulmonary arterial pressure increases.^8^ Abrupt increase in pulmonary vascular resistance results in right ventricular strain, changing the right ventricle (RV) myocardial contractile properties. As the pulmonary pressure increases, to maintain flow through the partially obstructed pulmonary vascular bed, the RV undergoes adaptations to cope with increased workload. However, the ability to adapt rapidly is limited.

The subsequent prolongation of RV contraction time leads to delayed systolic filling, RV chamber dilation, flattening of the interventricular septum and eventually leftward bowing of the interventricular septum if the pressure in the RV exceeds that of the LV.^8^ The development of right bundle-branch block can further exacerbate ventricular desynchronization. This cycle leads to reduced RV output and in turn left ventricular underfilling leading to reduction of cardiac output, contributing to systemic hypotension and haemodynamic collapse.^8^ Additionally, hypoxia because of ventilation/perfusion mismatch impacts left ventricular contractility. Raised right ventricular end diastolic pressure and raised right atrial pressures may also lead to opening of a patent foramen ovale and subsequently increase the risk of paradoxical thromboembolism due to an interatrial right to left shunt.

Hypoxia from PE is due to mechanical obstruction of the vascular bed and local cytokine release, which alters the ventilation to perfusion ratio. Additionally, inflammation results in surfactant dysfunction and atelectasis contributing to functional intrapulmonary shunting.^9^ Inflammation is also thought to be responsible for stimulating the respiratory drive that leads to hypocapnia and respiratory alkalosis.

### Clinical presentation

The clinical presentation of a PE can be highly variable but standardized nomenclature has been developed to improve communication and care, allowing a more streamlined approach to management. This includes the pattern of presentation, haemodynamic stability, anatomic location, and symptomology^10^ (Table 1).

**Table 1.** Patterns of clinical presentation in acute pulmonary embolism (PE).

Pattern of presentation	Acute—Symptoms and signs develop immediately after obstruction of pulmonary vessels. Sub-acute—within days or weeks following the initial event. Chronic—develop symptoms of breathlessness over many years
Anatomic location and distribution	Saddle—Embolus at the bifurcation of the main pulmonary artery, often extending into the right and left main pulmonary arteries. Lobar/Segmental/Sub segmental—Embolus distally within the main lobar, segmental, or sub segmental branches of a pulmonary artery. Distribution—PE can be bilateral or unilateral. Some also describe distribution as proximal and distal.
Symptoms	Symptomatic—symptoms leads to radiologic confirmation of PE Asymptomatic- Incidental finding of PE on imaging

The most common presenting symptom is dyspnoea followed by chest pain. This may mimic “cardiac pain” or “pleuritic pain” due to pleural irritation secondary to pulmonary infarction with distal disease. Haemoptysis is an unusual presenting symptom. Rarely patients present with shock, arrhythmia, or syncope. Several patients, including some with large PE, are asymptomatic or have mild or nonspecific symptoms. Accordingly, a high level of suspicion is required so that clinically relevant cases are not missed.^11^

An approach that selectively integrates clinical evaluation, pre-test probability assessment, PE rule out criteria (PERC), D-dimer testing, and imaging has been extensively studied in literature and forms the basis of current clinical practice.^12^

### Risk stratification of acute PE

Early mortality risk in acute PE can be stratified^1^ (Table 2). The presence of RV dysfunction is the main determinant of early clinical course and risk of mortality.^1^ Acute PE which is associated with circulatory shock or persistent hypotension has a mortality risk of 58% and is classified as high-risk (or massive PE by the American Heart Association).^11^ It accounts for 5% of acute PE patients.

**Table 2.** Stratification of patients with acute pulmonary embolism (PE) based on early mortality risk^1^.

Early mortality risk	Risk parameters and scores			
	Shock or hypotension	PESI class III-V or simplified PESI ≥1	Signs of RV dysfunction on imaging	Cardiac laboratory biomarkers
High	+	+	+	+
Intermediate-high	−	+	Both positive	
Intermediate-low	−	+	Either one (or none) positive	
Low	−	−	Assessment optional, if assessed both are negative	

Abbreviations: PESI = Pulmonary Embolism Severity Index; RV = right ventricle.

The challenge with the remaining cases is to determine which patients have low risk of early mortality and are thus suitable for outpatient management with oral anticoagulation, versus those that have an intermediate risk so require in-hospital treatment and monitoring. Patients with established co-morbidities for example coronary artery disease have prognostic implications in acute PE and as such play an important role when clinicians weigh up therapy options. A detailed discussion of co-morbidities is beyond the scope of this article.

The Pulmonary Embolism Severity Index (PESI) uses baseline clinical parameters to derive prediction scores about the course of acute PE (Table 3). PESI risk class I and II have a low risk of early mortality (<1.6% and 1.7–3.5%, respectively).^12^

**Table 3.** Original and simplified Pulmonary Embolism Severity Index (PESI)^1^.

Parameter	Original PESI	Simplified PESI
Age	Age in years	1 point (if age >80 years)
Male sex	+10 points	–
Cancer	+30 points	1 point
Chronic heart failure	+10 points	1 point
Chronic pulmonary disease	+10 points	
Heart rate ≥ 110 beats/min	+20 points	1 point
Systolic blood pressure < 100 mmHg	+30 points	1 point
Respiratory rate > 30 breaths/min	+20 points	–
Temperature <36°C	+20 points	–
Altered mental status	+60 points	–
Arterial oxyhaemoglobin <90%	+20 points	1 point

• Original PESI; Class I: <65 points (very low 30-day mortality risk of 0%-1.6%). Class II: 66–85 points (low mortality risk 1.7%–3.5%). Class III: 86–105 points (moderate mortality risk 3.2%–7.1%). Class IV: 106–125 points (high mortality risk 4.0%–11.4%). Class V: >125 points (very high mortality risk 10.0%–24.5%).

• Simplified PESI; 0 points = 30-day mortality risk 1.0%. ≥ 1 point = 30-day mortality risk 10.9%.

Acute PE patients not haemodynamically compromised but with a PESI class of III-V are classified as intermediate risk of early mortality. These patients can be further classified as intermediate-high risk of early mortality if they exhibit imaging signs of RV dysfunction (on echocardiogram or CTPA) and elevated biochemical markers of myocardial injury (elevated serum cardiac troponin I or T) or heart failure due to RV dysfunction (elevated B-type natriuretic peptide, NT-proBNP). These intermediate-high risk patients have a 21% mortality risk at 3 months.^24^ If only one or none of the imaging or biochemical markers of RV strain is present then the patient is classified as having intermediate-low risk of early mortality with a 2–3% 3-month mortality risk.^11^

## Imaging in acute pulmonary embolism

There are multiple non-invasive imaging modalities available to diagnose PE, including chest radiograph, computed tomography pulmonary angiography (CTPA), ventilation-perfusion (V/Q) scintigraphy (planar V/Q and single-photon emission computed tomography, SPECT V/Q), echocardiography, and magnetic resonance pulmonary angiography (MRPA). CTPA and V/Q scintigraphy are the 2 most widely used modalities. The choice of imaging modality used is determined by what can be performed in a timely manner. With numerous pathologies such as acute coronary syndrome or an aortic dissection with similar clinical presentations, it is paramount to acquire imaging as a priority. The practice of delaying imaging in haemodynamically stable patients seems outdated.

### Computed tomography pulmonary angiography

CTPA is widely performed as it is readily available even out of hours in most institutions, and it is quick when compared to other techniques. One of the main benefits of a CTPA is its capacity to evaluate pathology outside the pulmonary arteries, e.g. pneumonia, pneumothorax, rib fractures, heart failure, and acute aortic syndromes.

In addition to standard CTPA, there is potential for a “dual or triple rule out” in which the acute aortic syndrome and coronary arteries can be assessed in a single acquisition. However, due to the lack of adequately trained staff especially out of office hours, this is not yet standard practice in the UK.

**Figure 1.**
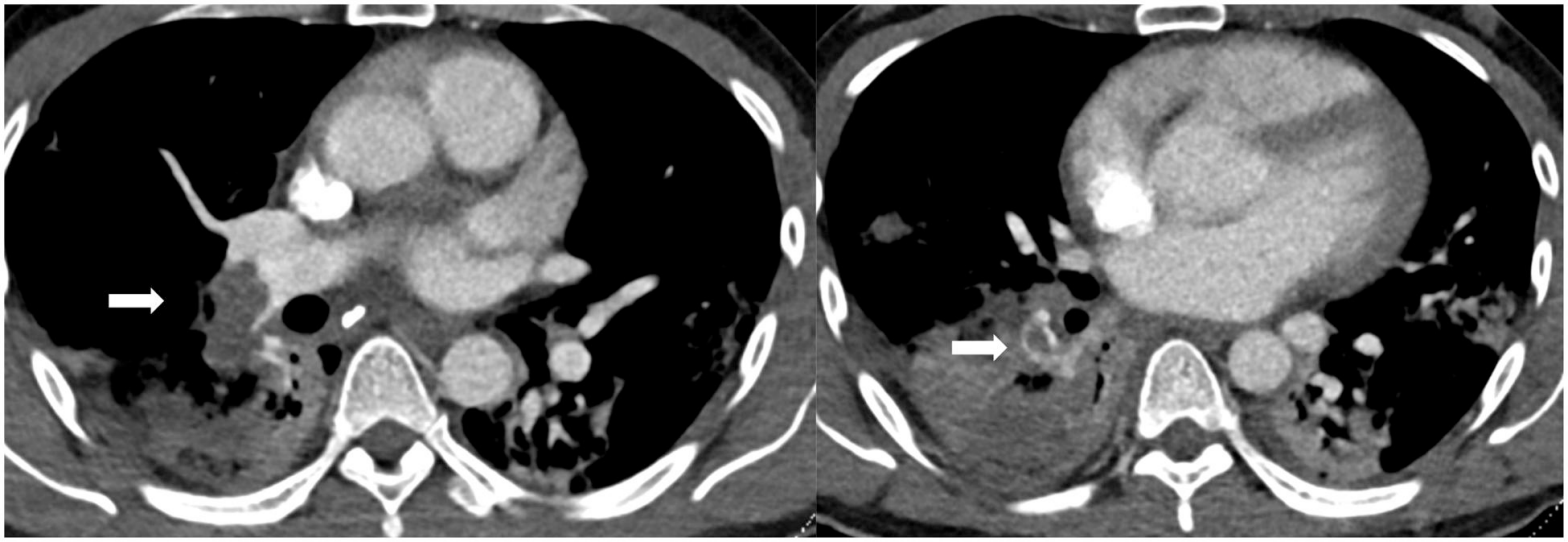
Filling defect in keeping with acute thrombus within the right lower lobe pulmonary artery. There is also bilateral lower lobe consolidation.

**Figure 2.**
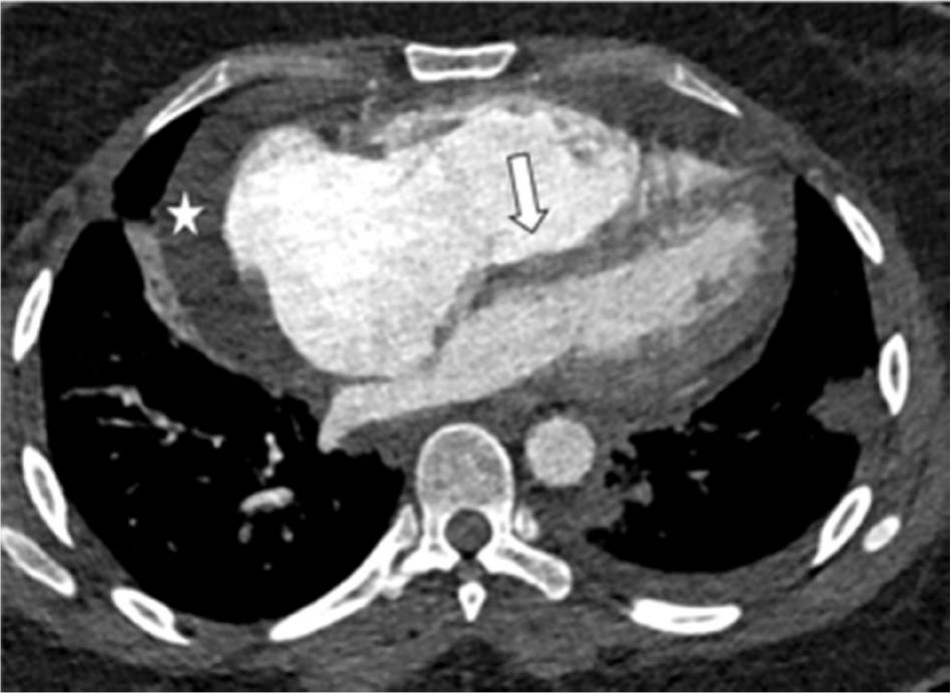
Right heart strain is demonstrated by a dilated right ventricle (RV), flattening and reversal of the interventricular septum (arrow). The right atrium is also dilated and bowing into the left atrium. There is a small pericardial effusion (star) and left pleural effusion. Small left subpleural consolidation is in keeping with an infarct.

**Figure 3.**
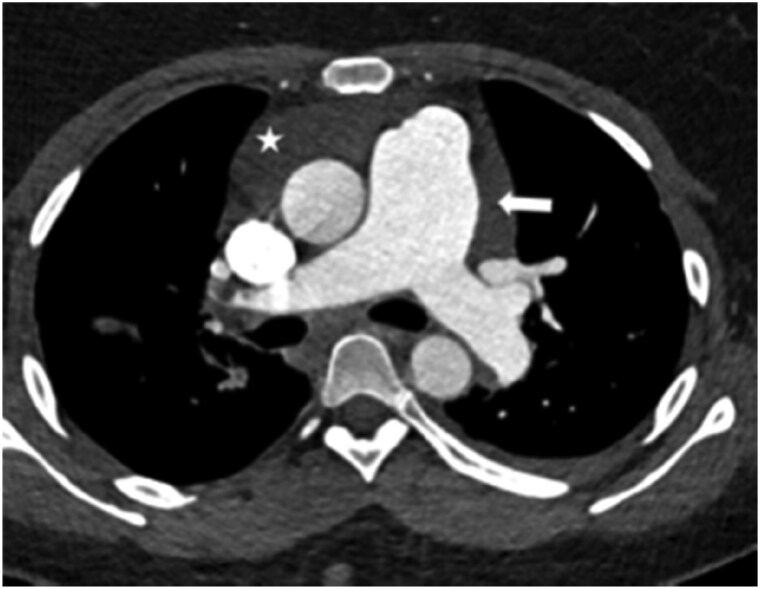
Main pulmonary artery (arrowhead) is dilated when compared to the ascending aorta at the same level. There is also a moderate volume of fluid in the pericardial recess (star).

Acute PE is directly visualized as central luminal filling defects within the opacified pulmonary arteries (Figure 1 and Supplementary Figure A). An accurate description of the pulmonary arterial branches involved and the degree of thrombus burden should be provided. RV strain on CTPA is assessed by RV dilation when compared to LV (RV: LV ratio > 1) on transverse images, flattening or leftward bowing of the septum on multiplanar reformatted images, and PA dilation^13^ (Figures 2 and 3 and Supplementary Figures B and C). PA dilation is measured at the level of the ascending aorta (AO) and a ratio of PA: AO >1 is considered abnormal. Truong et al. suggested using sex-specific normal cutoffs for pulmonary arter (PA) diameter in men (⩽29 mm) and women (⩽27 mm).^14^ Assessment of RV dilation on reformatted images (e.g. 4 chamber view) may be more accurate. Other signs of raised RV pressure include dilated inferior vena cava (IVC) and hepatic veins. Often reflux of contrast into IVC and hepatic veins is also seen.

The National Confidential Enquiry into Patient Outcome and Death (NCEPOD) recommends standardized CTPA reporting. This must include the assessment of RV strain. Approximately one-third of reports in UK did not comment on the RV in a recent audit.^15^ Since this information is crucial to risk stratification, the presence or absence of RV strain should be included in CTPA reports that are positive for PE (not necessary where CTPA is negative).

Sufficient enhancement of the pulmonary arteries on the imaging data set relies on rapid delivery of iodine-based contrast media into the PA timed to coincide with image acquisition targeted on the pulmonary arteries. Adequate contrast enhancement is generally accepted to equate to a density of > 250 Hounsfield Unit (HU) in the main pulmonary artery. An increased volume of contrast may be advantageous and help with adequate contrast enhancement in patients with larger body habits.

In addition, suboptimal CTPA may be related to patient motion, including breathing, large body habitus and beam hardening artefact from metal foreign bodies.^16^ Many patients unwittingly perform a Valsalva manoeuvre when given instructions to breath hold. This alters the intrathoracic pressure and causes transient interruption of contrast into the pulmonary arteries. In such cases, there may suboptimal contrast within the pulmonary arteries at automatic trigger, but higher density within the aorta and superior vena cava on the diagnostic scan. Therefore, coaching patients before image acquisition is helpful in improving image quality. Occasionally, an on-table assessment may be of value to improve image quality if the factors causing poor image quality can be mitigated on a second attempt. If a second acquisition is undertaken, then an expiratory volume or a free breathing study has been shown to improve diagnostic yield.^17^ The free breathing technique can also be used in mechanically ventilated patients, if the escorting team decides that the suspension of ventilation is unsafe.

As always, radiation exposure should be minimized, particularly in young and pregnant patients where tissues are more radiation sensitive. Most scanners can perform low kV studies; with the addition of iterative reconstruction the effective radiation dose can be as low as 0.9 mSv (this is well within the diagnostic reference levels for CTPA of 6.2 mSv or dose length product of 440 mGycm).^18^ In addition to reduced radiation exposure, low kV also improves apparent iodine contrast conspicuity.

Performing a CTPA is not always straightforward and obtaining diagnostic images in specific patient groups such as pregnant ladies or patient with a high cardiac output can prove to be challenging. This, however, can be mitigated to a degree by educating patients prior to image acquisition.

Once a PE has been diagnosed on a CTPA it is important to assess the degree of vascular occlusion to guide therapeutic decisions. Indicators to measure the severity of blockage on CTPA in addition to showing the existence of pulmonary emboli have been explored such as Quanadli index. The Quanadli index offers a relatively usable means of precisely assessing the degree of vascular obstruction.

CT venogram (CTV) of the lower extremities and pelvis to evaluate for DVT is not routinely performed concurrently with CTPA. However, when CTV is added to CTPA, it may marginally improve diagnostic yield.^19^ Assessment of the abdominal and pelvic veins may reveal a potential source of the acute PE.

At our institutions, extracorporeal membrane oxygenation patients have a full body CT from head to pelvis as part of the admission procedure. CTV of extremities can be considered at the discretion of the radiologist and clinical team.

### V/Q scintigraphy

Unlike CTPA, V/Q scintigraphy indirectly visualizes PE. The underlying principle is to identify a mismatch in the bronchopulmonary segments of normal ventilation but reduced perfusion. Ventilation sequences are usually acquired after inhalation of radioactive gas (^81m^Krypton) or radioaerosols e.g., technetium-labelled aerosol of diethylene triamine penta-acetic acid (^99m^Tc DTPA) and Technegas (^99m^Tc labelled carbon particles). There is no significant difference in overall accuracy between these two agents, and the choice varies between countries and institution.^20^ Ventilation sequences are prone to artefacts in restrictive pathologies such as asthma and chronic obstructive pulmonary disease. Unlike radioactive gases, radioaerosols do not wash out swiftly and hence allow for multiple image acquisitions. Perfusion sequences are usually acquired following the ventilation study, in order to achieve adequate count rates. Perfusion assessment requires an injection of ^99m^technetium labelled macro-aggregated human albumin (^99m^TcMAA) particles. These particles are confined to the pulmonary capillary bed during first pass and demonstrate the distribution of blood flow. V/Q scintigraphy can be performed either as planar V/Q or single-photon emission computed tomography V/Q_SPECT_+/− CT. Planar V/Q is a 2D acquisition of the lungs in different positions only, whereas V/Q_SPECT_is a 3D acquisition which can be coupled with an additional CT component of the chest on hybrid cameras. The ventilation component of a V/Q study can be omitted if perfusion study is normal, a strategy that may reduce radiation dose.

An advantage of planar V/Q when compared to CTPA is lower radiation dose. Nevertheless, planar V/Q studies is of lower resolution, and when performed on patients with an abnormal chest radiograph are more likely to result in false positives or indeterminate assessment.^21^

**Figure 4.**
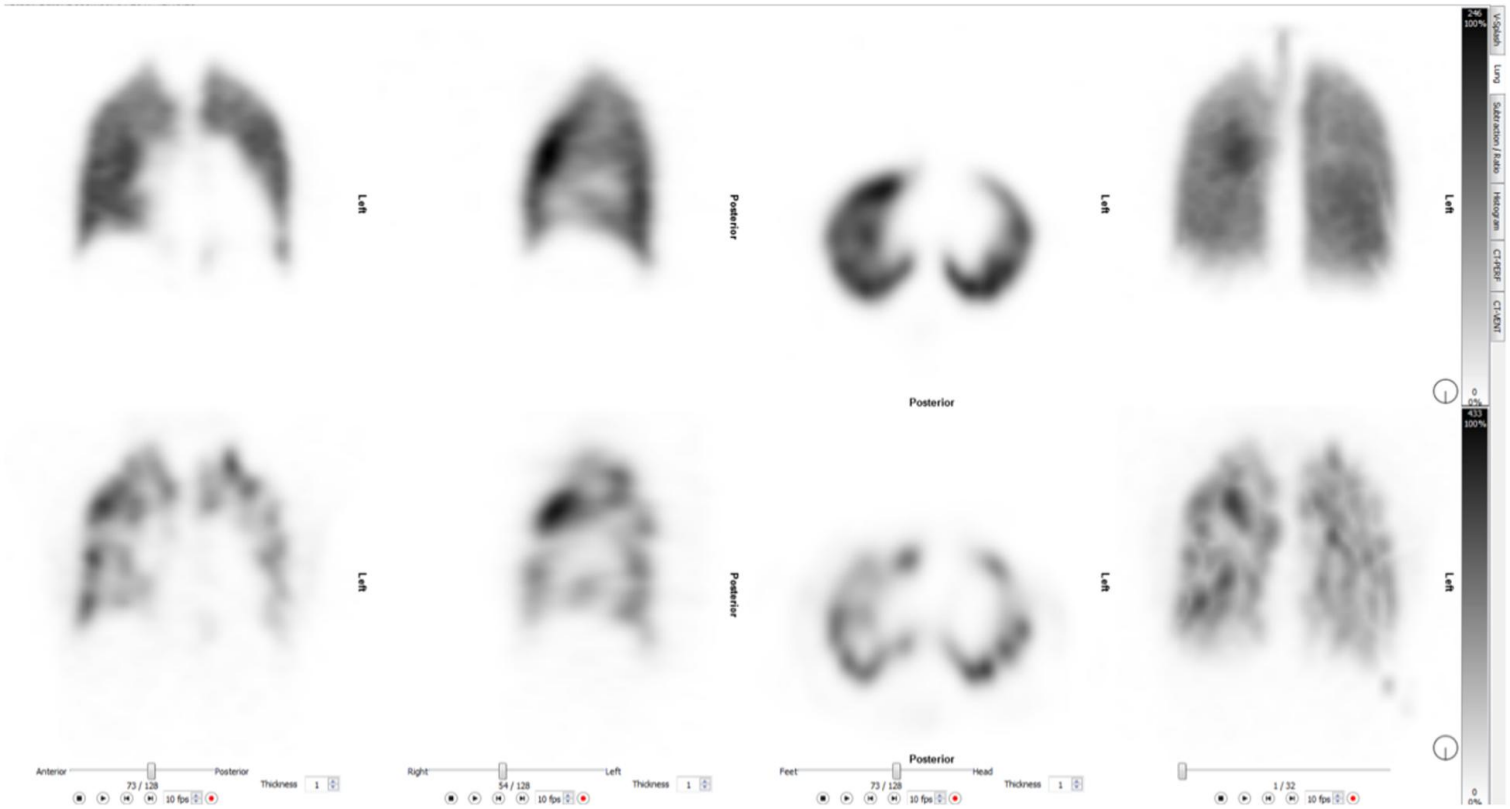
V/Q _SPECT_. Multiple bilateral subsegmental perfusion defects. Top row demonstrates ventilation with krypton, the bottom row demonstrates perfusion.

**Figure 5.**
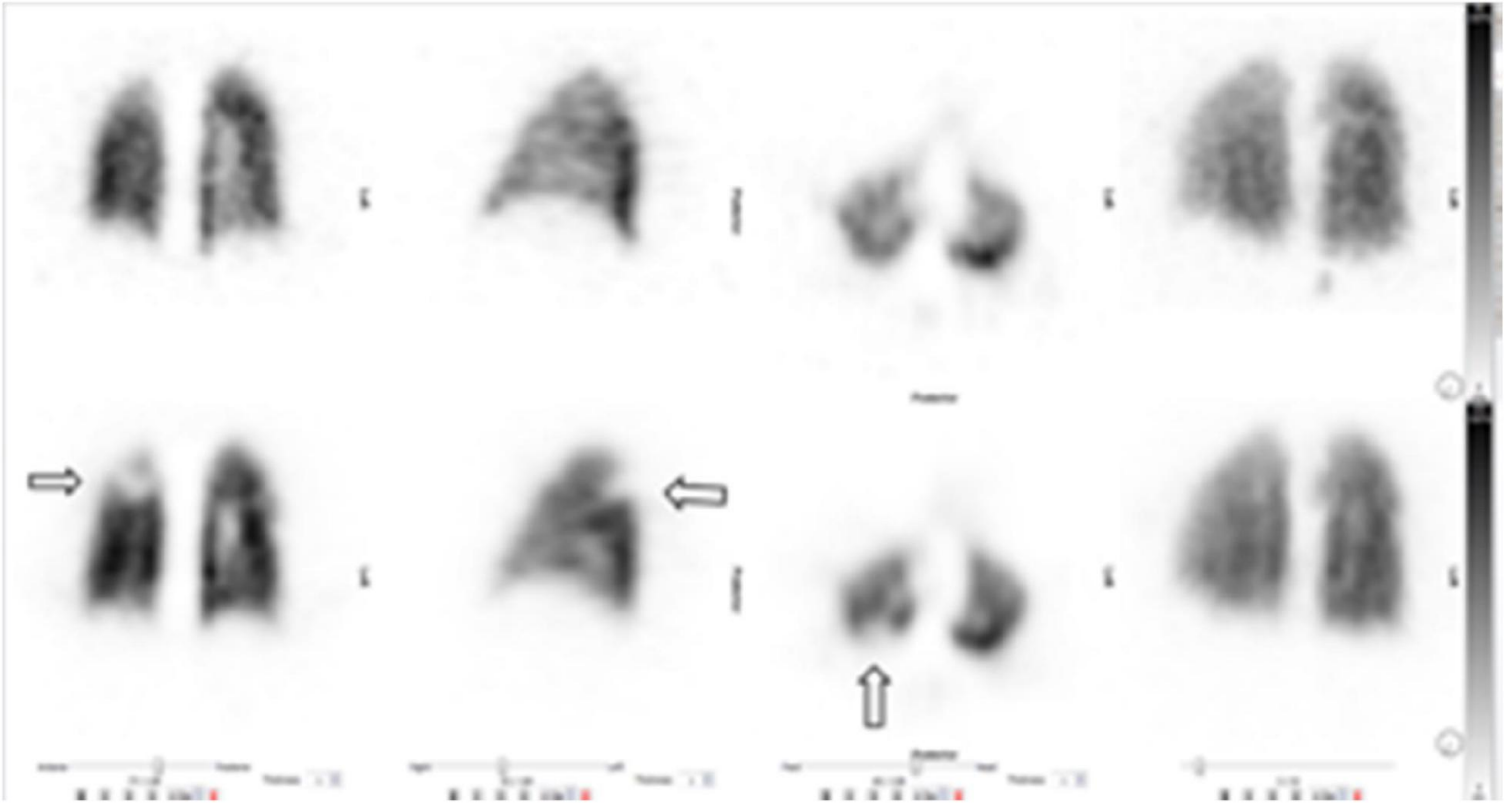
Right upper and lower lobe segmental perfusion defect (arrows).

The adoption of V/Q_SPECT_ has resulted in significant enhancement in imaging technology, enabling simultaneous assessment of the lung parenchyma, and identifying confounding conditions such as emphysema and pneumonia, both causes of false positive results of only perfusion studies. PE is reported as present when there is one segmental or more than one subsegmental mismatch^22^ (Figures 4 and 5).

V/Q_SPECT_ has very high sensitivity and specificity above 95%, whereas CTPA has a similar specificity but lower sensitivity of around 78%.^22^ Possible pitfalls of V/Q_SPECT_ include misalignment of the SPECT and CT images, or artefacts due to aggregation of ^99m^TcMAA particles.

The limitation of planar V/Q and V/Q_SPECT_ is availability only during “office hours”, particularly in the UK and is not readily available overnight and at weekends.

### Magnetic resonance pulmonary angiography

MRPA is an alternative to CTPA in specialized centres but is infrequently utilized in acute PE assessment. Although it avoids the use of ionizing radiation, it is less sensitive for acute PE, especially in the distal segmental and subsegmental pulmonary branches.^23^ MRPA utilizes gadolinium-based contrast which reduces the risk of nephrotoxicity and contrast allergies related to iodinated-contrasts used in CT. MRPA is also a longer examination than CTPA, rendering the technique unsuitable for acutely unwell patients. Some centres have reported use of gadolinium-based blood pool contrast agent MRPA and achieved sub 10 min studies. Overall, MRPA is less easily accessible, and could be considered suitable for young female patients in specialist centres.^24^ A potential advantage of MRPA is accurate “gold standard” assessment of cardiac function and dynamics.

### Chest radiograph

Chest radiographs are not sensitive or specific for diagnosing PE, and up to a quarter of radiographs can be normal. Common positive findings are cardiomegaly, pleural effusion, pulmonary artery enlargement, atelectasis, and pulmonary infiltrates.^25^

### Catheter pulmonary angiography

Catheter pulmonary angiography is an invasive technique that is usually reserved for patients where a concurrent therapeutic intervention is planned.

## Imaging in chronic pulmonary embolism

**Figure 6.**
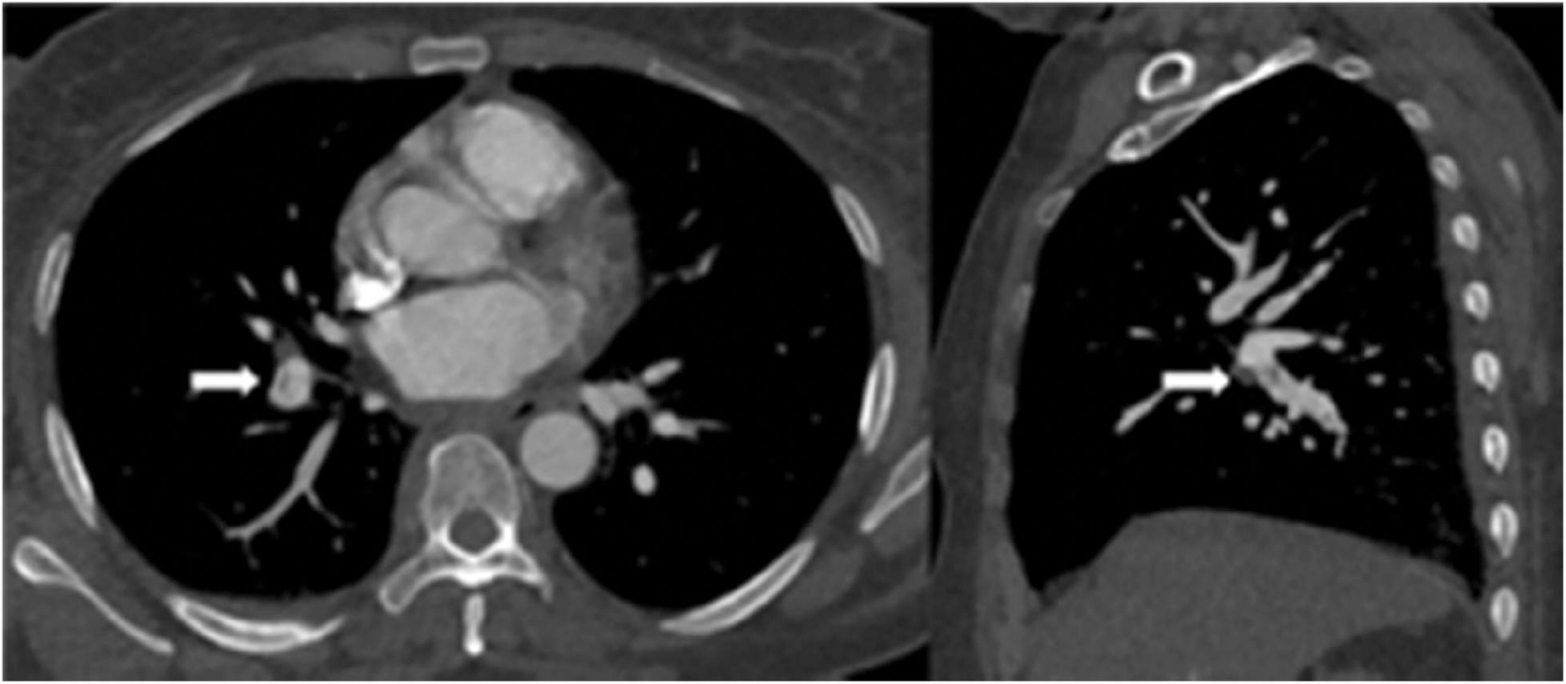
Axial and sagittal reconstruction of the right interlobar pulmonary artery (PA), demonstrating a linear filling defect in keeping with a web. (arrowhead).

After an acute episode of PE, some patients may not return to normal. They fall under 3 groups: those with CTED, CTEPH, or dyspnoea with functional limitations without identifiable pulmonary vascular disease (post-PE syndrome). CTED and CTEPH are the consequence of incomplete resolution of pulmonary emboli. The pathophysiology of non-resolution of thrombus is unclear. The residual thrombus organizes into fibrous tissue resulting in vessel stenoses, webs or occlusions (Figure 6 and Supplementary Figure D). Residual thrombus may calcify (Supplementary Figure E). In CTEPH, this chronic obstruction of the pulmonary arterial vasculature and subsequent microvascular remodelling results in raised pulmonary vascular resistance, leading eventually to right ventricular adaptive hypertrophy and potentially to RV failure. Pulmonary hypertension (PH) is defined as a resting mean pulmonary arterial pressure (mPAP) of ≥20 mmHg measured by invasive right heart catheterization.^26^ PH is classified into 5 groups as per the 2022 ESC/ERS Guidelines for the diagnosis of pulmonary hypertension, and CTEPH is a subtype of PH (group 4).

The imaging modalities used to diagnose chronic PE are largely the same as those used in acute PE assessment. In our experience, multi-modality imaging together with clinical assessment increases the diagnostic confidence of chronic PE. European Society of Cardiology recommend an initial echocardiogram and perfusion imaging (V/Q scintigraphy or other newer modalities like dual energy CTPA or MRI). A CTPA should also be performed, and supplemented by more invasive investigations like digital subtraction angiography and right heart catheteristiaon.^26^

### V/Q scintigraphy

A negative V/Q_SPECT_ is a powerful modality to exclude CTEPH confidently. It is thought traditionally to have a higher sensitivity than standard CTPA, especially for smaller distal PE. However, a more recent study using invasive angiogram as the gold standard, found both planar V/Q and V/Q_SPECT_ techniques more sensitive but less specific than CTPA^27^ Sensitivities and specificities may vary further among institutions depending on experience. Some institutions use a reduced dose of MAA in the context of pulmonary hypertension, as there is a theoretical risk of exacerbation of symptoms.^28^

### Computed tomography pulmonary angiography

Recognition of CTED and CTEPH on CTPA can be challenging. A small study in a specialist centre quoted the sensitivity of CTEPH from outside referrals to be as low as 26%.^29^ It is important to accurately report the location of acute or chronic PE, as proximal disease could be amenable to surgical endarterectomy, whereas more distal disease is usually treated medically. Disease predominantly in middle calibre vessels maybe amenable to balloon pulmonary angioplasty (BPA). Another sign of CTEPH is mosaicism of the lung parenchyma, due to areas of hypo and hyperperfusion.

**Figure 7.**
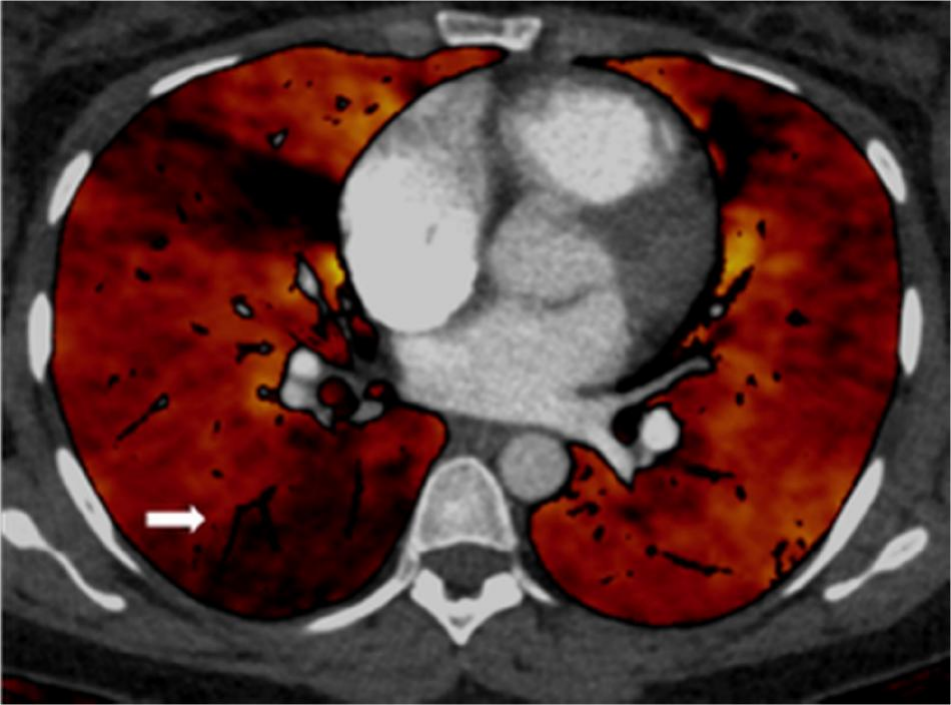
Perfused blood volume (PBV) map in a chronic thromboembolic pulmonary hypertension (CTEPH) patient. Wedge like areas of low PBV signal in the right middle lobe and right lower lobe from occluded segmental vessels.

Historically V/Q was the modality to assess perfusion distribution in the lungs. In recent times, however, DE-CTPA has become a strong competitor to provide more physiological information of the lungs. DE-CT is a technique that has existed since 1976 but has become more common place in recent years with advancement in technology.^30^ The technique can be achieved by scanning the patient with 2 separate beams, commonly a high (140–150 kVp) and low energy (80–100 kVp). “Dual energy” is somewhat of a misnomer, as the energy beams consist of a spectrum of energy levels, and hence sometimes known as “spectral CT”. DE-CTPA can be achieved by dual or single source techniques. A dual source CT has 2 X-ray tubes producing 2 beams of different energy levels. Similar data can be achieved by a single source beam with fast kV switch, dual layer detectors, or dual acquisition. The principle is to acquire 2 datasets of the same anatomy at different energy levels. Lung subtraction iodine mapping produce similar surrogate maps of perfusion from single energy scans, by subtracting pre-contrast scans from post-contrast using non-rigid registration techniques. It is a software technique only solution, and has shown some early correlation with SPECT V/Q. Since different materials absorb radiation in relation to atomic mass, post processing software may generate material specific and virtual monoenergetic images. In the context of CTPA, iodine distribution images can be generated for the lungs. This technique can be used in both acute and chronic PE. In CTED and CTEPH, the iodine mapping obtained from material decomposition may show segmental areas of reduced uptake in keeping with a reduced perfused blood volume (PBV) (Figure 7 and Supplementary Figures F and G). The sensitivity and specificity of Dual energy (DE)-CTPA approach that of V/Q_SPECT_ at 88.9% and 94.6%, respectively.^30^ DE-CTPA also has the benefit of visualizing abnormal pulmonary parenchyma, which can cause diagnostic difficulties in V/Q techniques. Novel techniques involving Xenon enhanced DE-CTPA has been demonstrated to provide functional information, but this is currently still under active research with limited clinical application.

### Magnetic resonance pulmonary angiography

**Figure 8.**
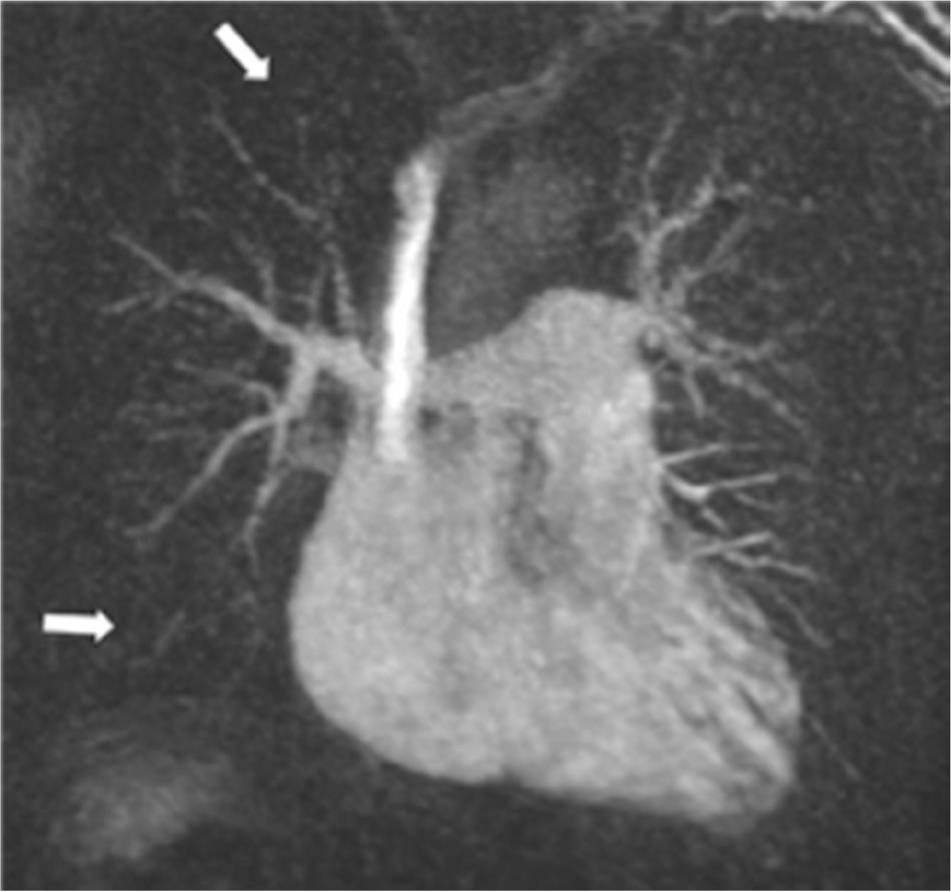
3D coronal maximum intensity projection (MIP) image of a magnetic resonance pulmonary angiography (MRPA) in a patient with chronic thromboembolic pulmonary hypertension (CTEPH). There is a lack of right lower lobe pulmonary artery vasculature. There is also reduction of the right upper lobe distal pulmonary vasculature. (arrowhead).

**Figure 9.**
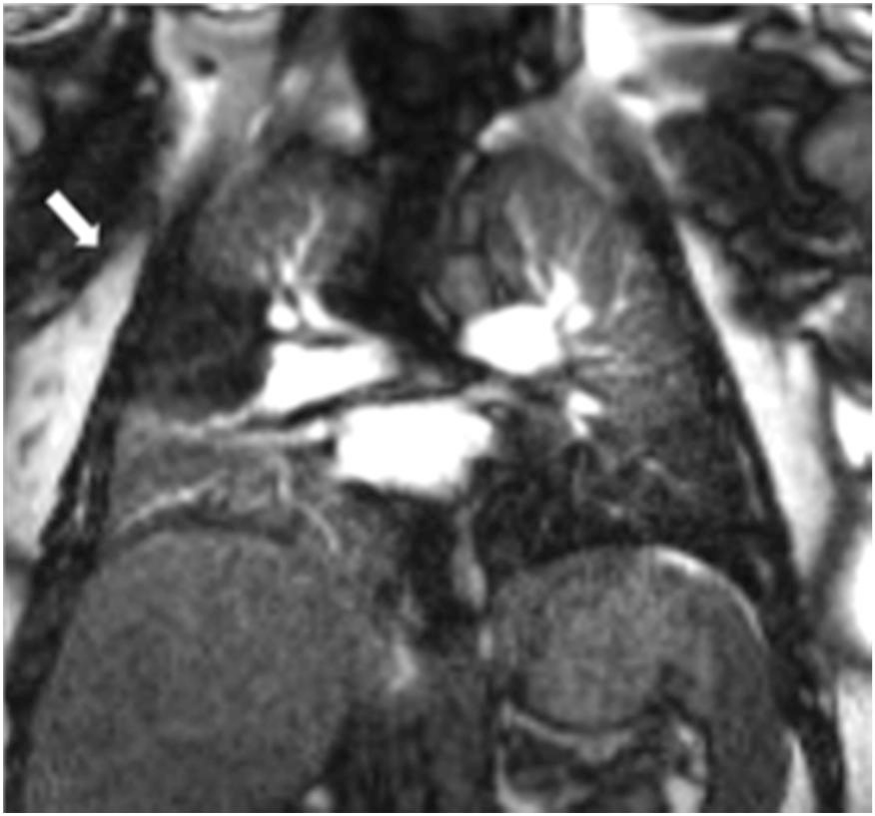
Right upper lobe wedge like decreased perfusion, which fills in later than the rest of the other pulmonary parenchyma.

Structural changes associated with CTED and CTEPH may also be demonstrated with MRPA (Figure 8), although the spatial resolution is lower than that achieved with CT, making it less sensitive to eccentric chronic PE when the vessel wall is difficult to visualize. Interestingly, recent literature has suggested MRPA might even be superior to CTPA for subsegmental pulmonary embolism, although there are limitations to the metanalysis.^32^ A 3D volume acquisition during contrast enhancement perfusion MRI can be performed as part of magnetic resonance pulmonary angiography MRPA protocol, providing dynamic information on speed of contrast delivery in the lungs, together with segmental perfusion abnormalities from first pass to washout (Figure 9). This technique has sensitivity and specificity like that of V/Q_SPECT_ for detecting chronic PE.^31^ It is important to correlate the areas of reduced perfusion with the pulmonary parenchyma on CT, as other pathology such as emphysema or fibrosis can also cause a perfusion defect. New on the horizon is 4D flow MRA which evaluates vortical blood flow which typically needs extensive post processing time. This is now starting to make transition from research to clinical use, and has potential to non-invasively predict PA pressures which will complement invasive imaging in patients.^33^

**Figure 10.**
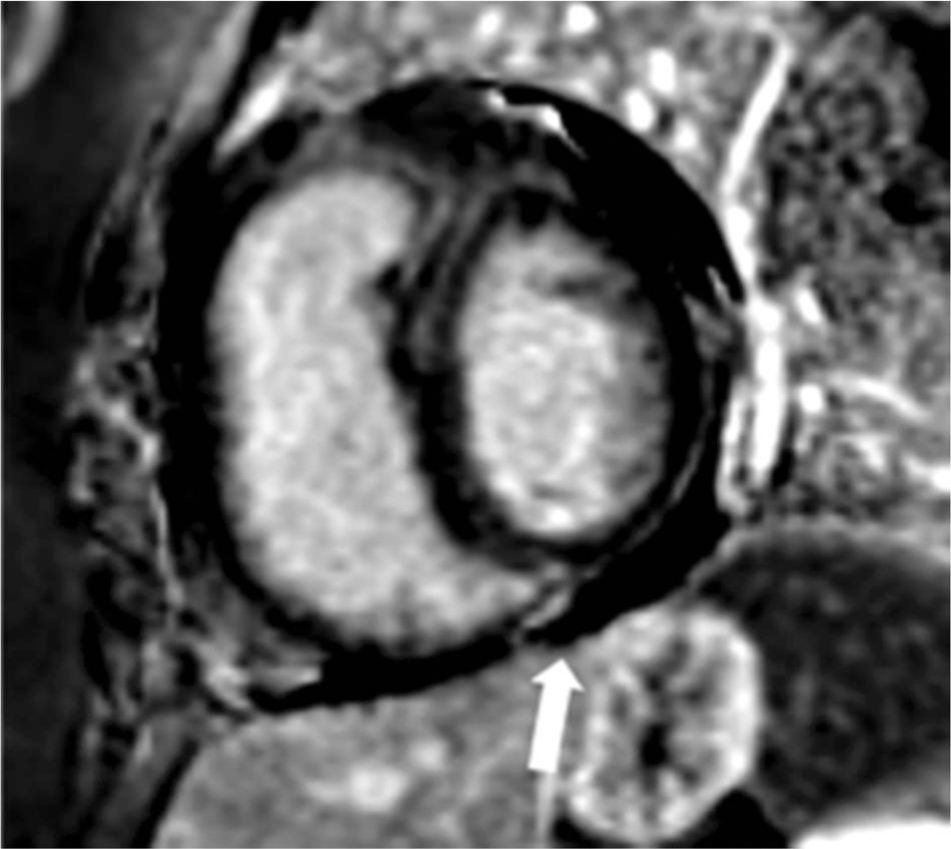
LGE of the inferior insertion point in a patient with CTEPH (arrow).

Cardiac MRI assessment may also be performed at the same time as MRPA as “gold standard” to assess cardiac function and dynamics. Features of right heart strain are demonstrated dynamically. It also provides additional information by demonstrating other signs which have unfavourable prognostic implications in the context of CTEPH. RV fibrosis can commonly be seen in the RV insertion points as late gadolinium enhancement on dedicated sequences and is thought to be a maladaptive response to pressure overload in right heart failure (Figure 10). It is more common in dilated RVs and associated with poorer RV function.^34^

The imaging strategy for chronic thromboembolism varies between institutions and depends on local expertise and technology available. Despite guidelines, it can still be a complex and dynamic process. At the moment, a normal V/Q_SPECT_ is a powerful tool in excluding CTED/CTEPH and may save the patient from further complex and time-consuming tests. However, DE-CTPA is a rapidly improving diagnostic test. Photon counting CT is promising in providing better spectral sensitivity and ultra high resolution of the lungs. This may help to further phenotype patients with PH. MRPA combined with CMR is also an advancing technology on the horizon. Although MR cannot fully replace V/Q SPECT and DE-CTPA in the workup at the moment, its lack of radiation is a huge advantage for repeat studies especially in the younger population. It also has an advantage of providing functional information and prognosis. 4D MRI is an emerging technique which demonstrates wall shear stress of pulmonary arteries and measures stiffness and distensibility,^35^ and is promising as an alternative non-invasive method of measuring haemodynamics. However, it is currently only available in a select few specialist centres. cardiac MRI (CMR) PET is also only available in niche specialist centres, but its ability to combine RV metabolism and haemodynamics is promising for further prognostication.^36^ CombinedCMR and CPET (cardiopulmonary exercise testing) aims to provide information on both cardiac output and oxygen consumption, and maybe helpful in prognostication of systemic sclerosis patients.^37^ Artificial intelligence has the potential to rapidly process cross sectional images to provide fully automated diagnosis of PH with volumetry, obviating the need for manual segmentation.^38^ It can also increase the detection rates of PEs.^39^

It is important to note, however, that in spite of vast improvements in non-invasive technology, most CTEPH patients currently still undergo right heart catheterization as gold standard.

## Conclusion

PE is a common medical presentation that requires integration of clinical and imaging expertise to reduce morbidity and mortality. With improving imaging technology and post processing ability, multiple non-invasive imaging modalities have gained prominence in the diagnosis and prognostication of patients with both acute and chronic PE. The integrated use of these modalities improves the diagnostic confidence in managing this condition.

Owing to the review nature of this article, Institutional Review Board approval was waived.

## Supplementary Material

tzaf005_Supplementary_Data
