# Supplementary material for: Non-invasive imaging in acute and chronic pulmonary embolism
Source: BJR Open. 2025 Apr 10;7(1):tzaf005. doi: 10.1093/bjro/tzaf005 (PMC12254125; doi:10.1093/bjro/tzaf005)
Supplement: tzaf005_Supplementary_Data [file tzaf005_supplementary_data.zip › Supplementary_Data/Supplementary_Data/Supplementary figures captions.docx]

Supplementary figures

A Filling defects within the right lower lobe segmental and subsegmental pulmonary

arteries (arrows).

B Dilated IVC and hepatic veins (arrow) due to increased right sided pressure. There is also a

small volume of ascites in keeping with decompensated heart failure.

C Axial balanced steady state free precession MRI image of a patient with occlusive left

common iliac vein thrombus (arrow).

D CTPA of a patient with CTEPH. There is complete lack of enhancement in the right lower lobe. Note the reduced but presence of left lower lobe vascularity (arrow). There is also right ventricular dilatation and interventricular septum flattening.

E Chronic PE in the main pulmonary arteries with peripheral calcification.

F Mosaicism of the lung parenchyma in a patient with known CTEPH. The “darker” lungs

are due to decreased perfusion, whereas the “whiter” lungs are in keeping with increased

perfusion.

G Right lower lobe subsegmental acute PE (arrow), with a corresponding reduced area of pulmonary blood volume signal (star).
