# Supplementary figures and images for: Non-invasive imaging in acute and chronic pulmonary embolism
Source: BJR Open. 2025 Apr 10;7(1):tzaf005. doi: 10.1093/bjro/tzaf005 (PMC12254125; doi:10.1093/bjro/tzaf005)

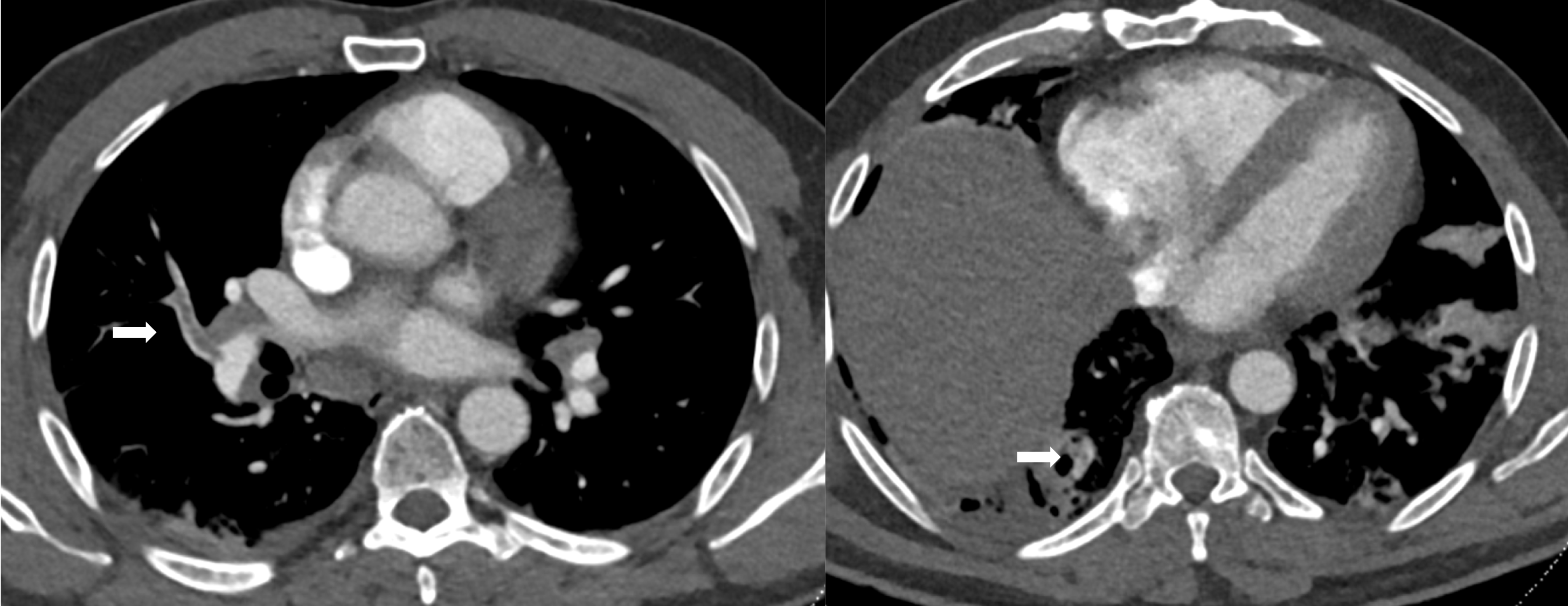

Supplement: tzaf005_Supplementary_Data [file tzaf005_supplementary_data.zip › Supplementary_Data/Supplementary_Data/Supplementary fig A.png]

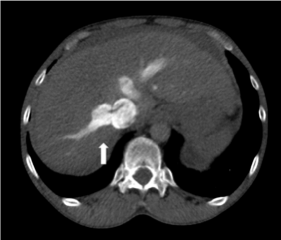

Supplement: tzaf005_Supplementary_Data [file tzaf005_supplementary_data.zip › Supplementary_Data/Supplementary_Data/Supplementary fig B.png]

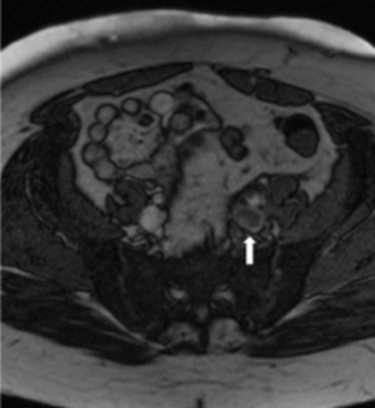

Supplement: tzaf005_Supplementary_Data [file tzaf005_supplementary_data.zip › Supplementary_Data/Supplementary_Data/Supplementary fig C.png]

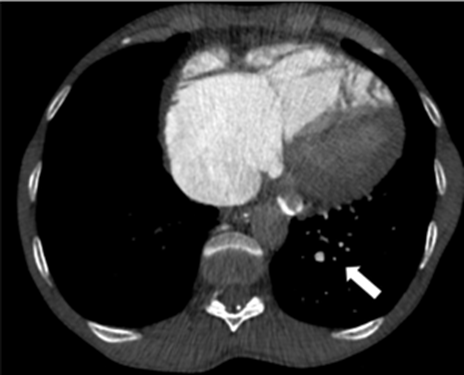

Supplement: tzaf005_Supplementary_Data [file tzaf005_supplementary_data.zip › Supplementary_Data/Supplementary_Data/Supplementary fig D.png]

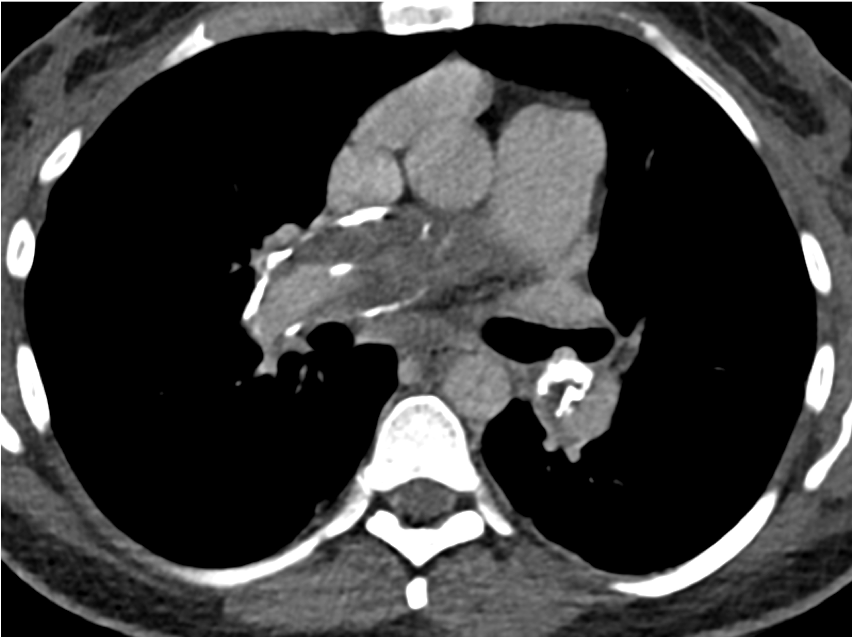

Supplement: tzaf005_Supplementary_Data [file tzaf005_supplementary_data.zip › Supplementary_Data/Supplementary_Data/Supplementary fig E.png]

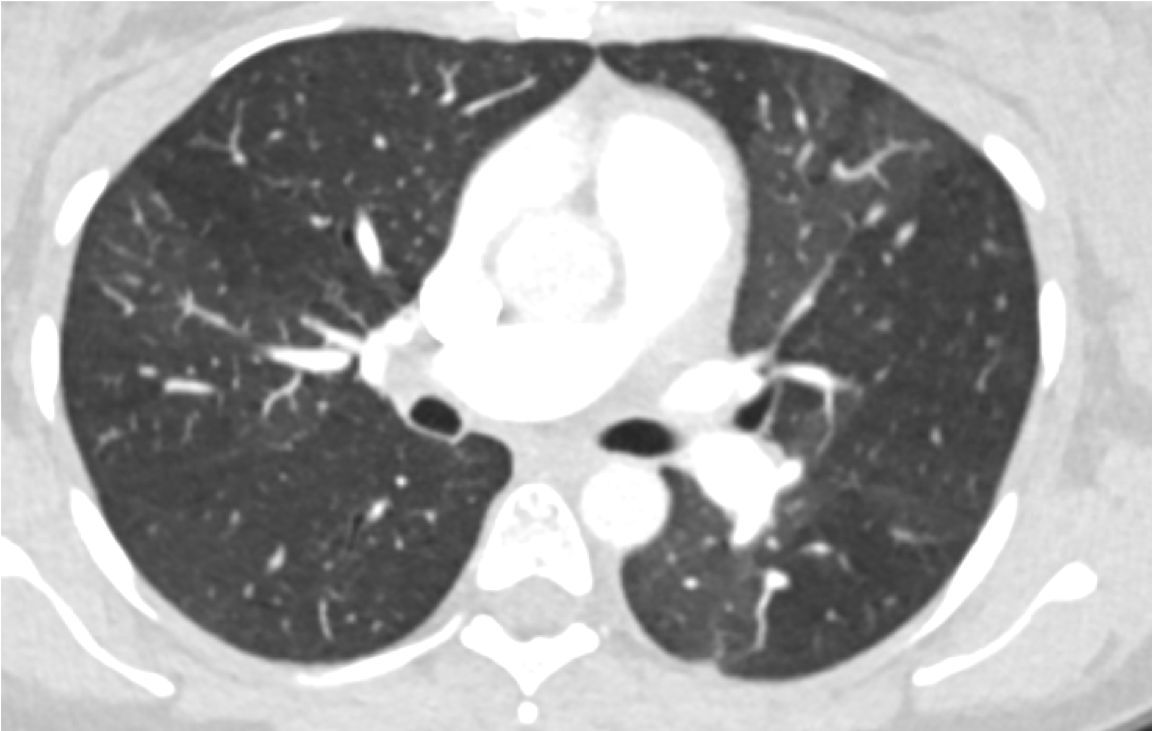

Supplement: tzaf005_Supplementary_Data [file tzaf005_supplementary_data.zip › Supplementary_Data/Supplementary_Data/Supplementary fig F.png]

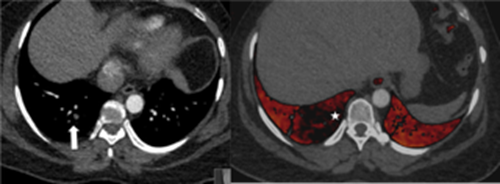

Supplement: tzaf005_Supplementary_Data [file tzaf005_supplementary_data.zip › Supplementary_Data/Supplementary_Data/Supplementary fig G.png]
